# Supplementary material for: Draft Aphaenogaster genomes expand our view of ant genome size variation across climate gradients
Source: PeerJ. 2019 Mar 11;7:e6447. doi: 10.7717/peerj.6447 (PMC6417409; doi:10.7717/peerj.6447)
Supplement: Supplemental Information 2 — NCBI genome database accession information for the previously sequenced ant genomes and coordinates for species those species that could be obtained from the published literature. [file peerj-07-6447-s002.pdf]

	Lon	Lat
Acromyrmex echinatior	9.12	-79.70
Atta cephalotes	9.12	-79.70
Atta colombica	9.12	-79.70
Camponotus floridanus	24.62	-81.54
Cardiocondyla obscurior	-19.92	-43.94
Cyphomyrmex costatus	9.12	-79.70
Dinoponera quadriceps	9.40	-79.87
Formica selysi	46.58	10.32
Harpegnathos saltator	15.32	75.71
Lasius niger	55.76	37.62
Linepithema humile	37.26	-122.02
Ooceraea biroi	26.21	127.68
Pogonomyrmex barbatus	20.59	-100.39
Pseudomyrmex gracilis	-11.77	-70.81
Solenopsis invicta	33.95	-83.36
Trachymyrmex cornetzi	9.12	-79.70
Trachymyrmex septentrionalis	30.44	-84.28
Trachymyrmex zeteki	9.12	-79.70
Vollenhovia emeryi	20.59	-100.39

Table 1: Coordinates obtained from the literature published for the ant genome sequences used in the biogeographic analyses.
