# Supplementary material for: Draft Aphaenogaster genomes expand our view of ant genome size variation across climate gradients
Source: PeerJ. 2019 Mar 11;7:e6447. doi: 10.7717/peerj.6447 (PMC6417409; doi:10.7717/peerj.6447)
Supplement: Supplemental Information 4 — PerMANOVA pseudo-F table for the analysis of the factors correlated with ant genome size only including the previously sequenced NCBI ant specimens. [file peerj-07-6447-s004.pdf]

	df	*SS*	*MS*	*Pseudo-F*	*R2*	p-*value*
Size Distance						
Lat	1	2707.43	2707.43	2.11	0.07	0.1796
Lon	1	1759.79	1759.79	1.37	0.05	0.2693
MAT	1	118.64	118.64	0.09	0.00	0.7636
Tmin	1	3394.10	3394.10	2.65	0.09	0.1434
Tmax	1	5518.63	5518.63	4.31	0.14	0.0727
PA	1	8349.14	8349.14	6.52	0.21	0.0363
PS	1	5501.51	5501.51	4.29	0.14	0.0679
Residuals	9	11533.39	1281.49		0.30	
Total	16	38882.63			1.00	
MASH Distance						
Lat	1	0.02	0.02	1.66	0.08	0.0683
Lon	1	0.02	0.02	2.07	0.10	0.0295
MAT	1	0.02	0.02	1.95	0.10	0.0332
Tmin	1	0.01	0.01	1.06	0.05	0.3679
Tmax	1	0.01	0.01	1.43	0.07	0.1483
PA	1	0.01	0.01	1.38	0.07	0.1590
PS	1	0.02	0.02	1.56	0.08	0.0871
Residuals	9	0.09	0.01		0.45	
Total	16	0.19			1.00	

Table 1: PerMANOVA pseudo-F table for the analysis of the factors correlated with ant genome size and similarity (MASH distance) only including the previously sequenced NCBI ant specimens.
