# Supplementary figures and images for: Draft Aphaenogaster genomes expand our view of ant genome size variation across climate gradients
Source: PeerJ. 2019 Mar 11;7:e6447. doi: 10.7717/peerj.6447 (PMC6417409; doi:10.7717/peerj.6447)

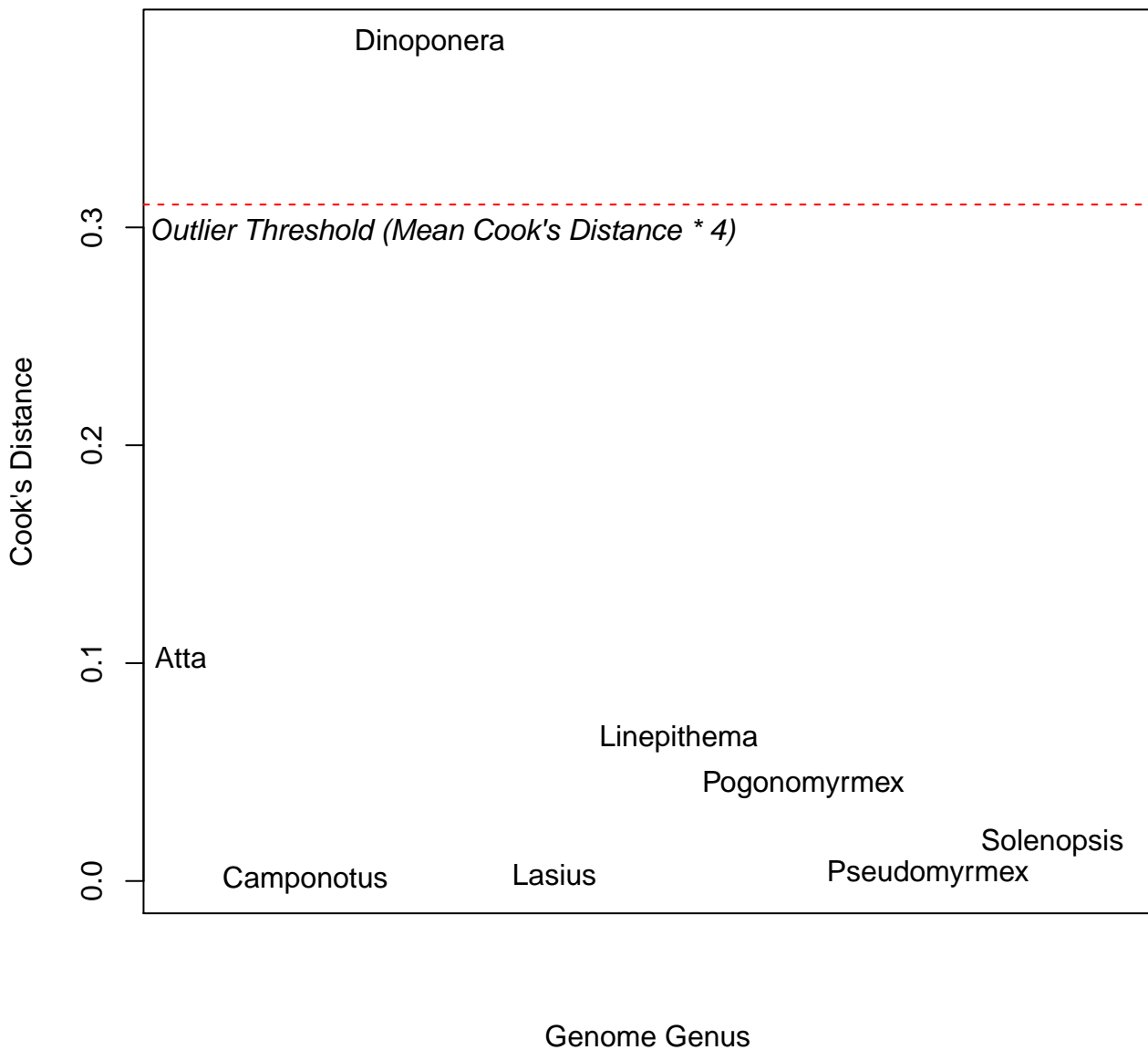

Supplement: Supplemental Information 5 — Outlier detection showing that Dinoponera quadriceps falls above the threshold for outliers, in this case 4 times the mean Cook’s distance from the regression of assembly length and genome size based on flow cytometry. [file peerj-07-6447-s005.pdf]
